# Acute kidney injury secondary to overtreatment of hypertension in an elderly patient undergoing combined cardiovascular surgery: A case report

**DOI:** 10.3892/mi.2026.316

**Published:** 2026-04-16

**Authors:** Saime Paydas, Alpay Turan Sezgin

**Affiliations:** 1 Department of Internal Medicine Nephrology, Acibadem Hospital Döşeme, Adana 01130, Turkey; 2 Department of Cardiology, Acibadem Hospital Döşeme, Adana 01130, Turkey

**Keywords:** acute kidney injury, hypertension, elderly patient, cardiovascular disease, surgery

## Abstract

The present case report describes the case of a 76-year-old diabetic female patient operated on for ischemic heart disease and aortic valve stenosis. The patient suffered from acute kidney injury related to hypotension secondary to the use of anti-hypertensive drugs. The present case report also discusses the need for greater caution in treating hypertension in elderly patients with diabetes and cardiovascular disease, drawing on the available literature. Patients should be encouraged to measure their blood pressure at home, and written blood pressure target values should be provided to both the patient and their caregivers. It is important to note that hypotension, similar to hypertension, may lead to complications such as falls, acute kidney injury and hospitalization. Furthermore, the adverse events that develop following simultaneous coronary artery bypass graft and surgical aortic valve replacement surgery in the elderly are discussed in an aim to provide further insight on this matter.

## Introduction

Arterial hypertension is a major modifiable risk factor for cardiovascular morbidity and mortality. The prevalence of hypertension, particularly systolic hypertension, increases with age. Due to arterial stiffening and frailty in older patients, the careful management of hypertension is essential (1). Older patients with hypertension have an increased vulnerability to developing adverse events. For this reason, it is recommended that the Systemic Coronary Risk Evaluation Older Persons (SCORE2-OP) system be used in clinical practice in the follow-up period to manage older patients with hypertension who are not already at a high or very high risk (2). The appropriate management of blood pressure in this population is a priority for clinicians. The administration of anti-hypertensive drugs should always take into consideration the benefits and risks associated with treatment, such as the development of adverse drug events, such as acute kidney injury (AKI), hypotension, falling, hyperkalemia, etc (3,4). In addition to cardiovascular disease risk stratification, a comprehensive geriatric-based evaluation is essential to guide management decisions regarding these older adults, since individualized decisions are often required (1).

In the American Heart Association 2025 hypertension guideline, the authors draw attention to how, among middle-aged and older adults, the use of intensive vs. standard blood pressure targets has increased the risk of developing adverse events in patients with orthostatic hypotension (OH). OH has been reported at rates of 7-10% in hypertensive adults, particularly among those who are older. OH is also associated with the use of anti-hypertensive drugs. It is critical to screen for OH prior to initiating or intensifying anti-hypertensive treatment, and to monitor for hypotension during treatment (5).

The management of coronary artery disease in older patients requires a complex and multidisciplinary approach. In addition to complications related to surgery, malnutrition and poor physical performance also affect patient prognosis (6). The present case report describes the case of patient with AKI related to the use of anti-hypertensive drugs who was operated on for ischemic heart disease and aortic valve stenosis.

## Case report

A 76-year-old diabetic female patient was admitted with fatigue to the nephrology outpatient clinic of Adana Acibadem Hospital on May 14, 2025. The patient experienced hypotension (systolic blood pressure, 80 mmHg), dizziness, weakness and had fallen to the ground at home. At that time, there was no history of loss of consciousness, weakness or vomiting. Large ecchymoses developed at the periorbital and gluteal regions, and legs. While her systolic blood pressure was measured at 80 mmHg, in the following days, she had continued to use anti-hypertensive drugs. She was hospitalized due to abnormal renal function tests, a blood urea nitrogen (BUN) level of 144 mg/dl and a creatinine level of 10.30 mg/dl on day 8 after the fall and hypotensive attack. She denied any changes in urine volume and color. Abnormal findings upon a physical examination were low blood pressure (80/60 mmHg in a sitting and standing position), ecchymoses at the periorbital regions and legs, arrhythmic heart sounds, prosthetic heart valve sound, jugular venous distension, positive hepatojugular reflux and pretibial edema.

The medical history of the patient included diabetes mellitus (DM), hypertension, and coronary artery bypass graft (CABG) and surgical aortic valve replacement (SAVR).

### Surgical procedure

Simultaneous CABG which involved the autogenous vein graft repair of five veins (internal mammary artery and saphenous vein), as well as SAVR (biological valve; a no. 23 Edwards valve was surgically implanted).

### Post-operative course and complications

During the early post-operative period, no complications developed. On post-operative day 10, the patient was discharged from the hospital. Cordarone (amiadorone) at 2x200 mg/day, cilazapril at 5 mg, hydrochlorothiazide at 12.5 mg (Inhibace Plus 5/12.5), warfarin at 1x5 mg/day, furosemide (Lasix tab) and insulin were prescribed. On post-operative day 34, a hematoma and abscess that had developed at the lower end of the sternum were drained and metoprolol (2x50 mg/day) was commenced.

### Findings, management and follow-up

**Figure 1:**
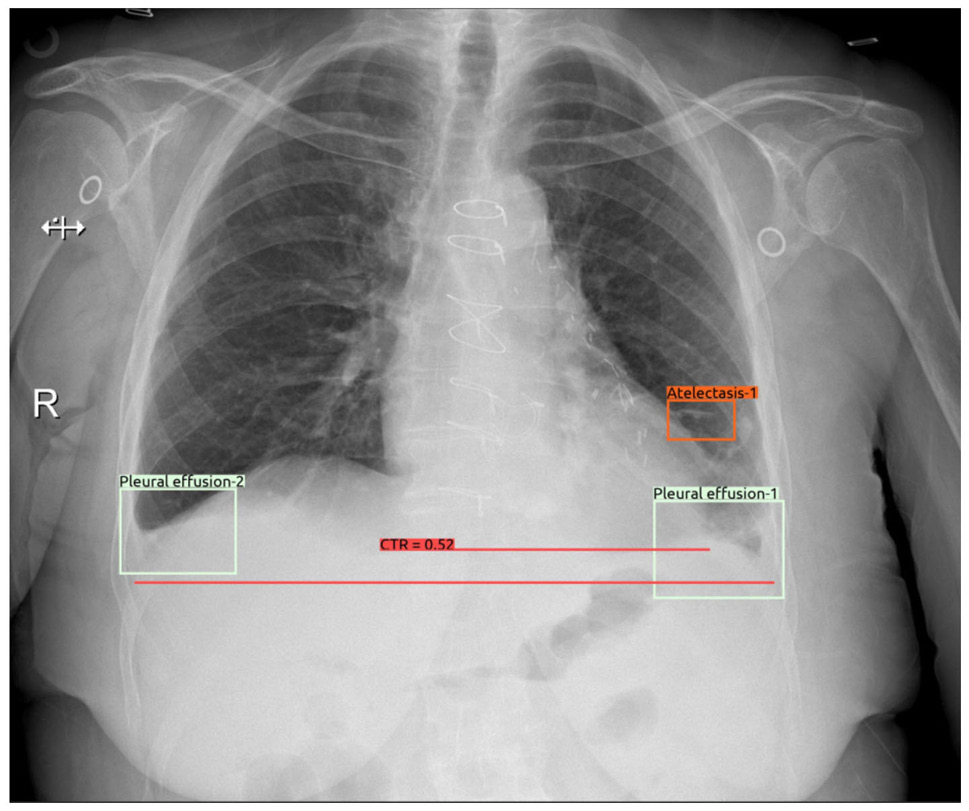
Bilateral pleural fluid, cardiomegaly and metallic suture and clip secondary to coronary artery bypass graft and surgical aortic valve replacement were determined in the chest X-ray.

**Figure 2:**
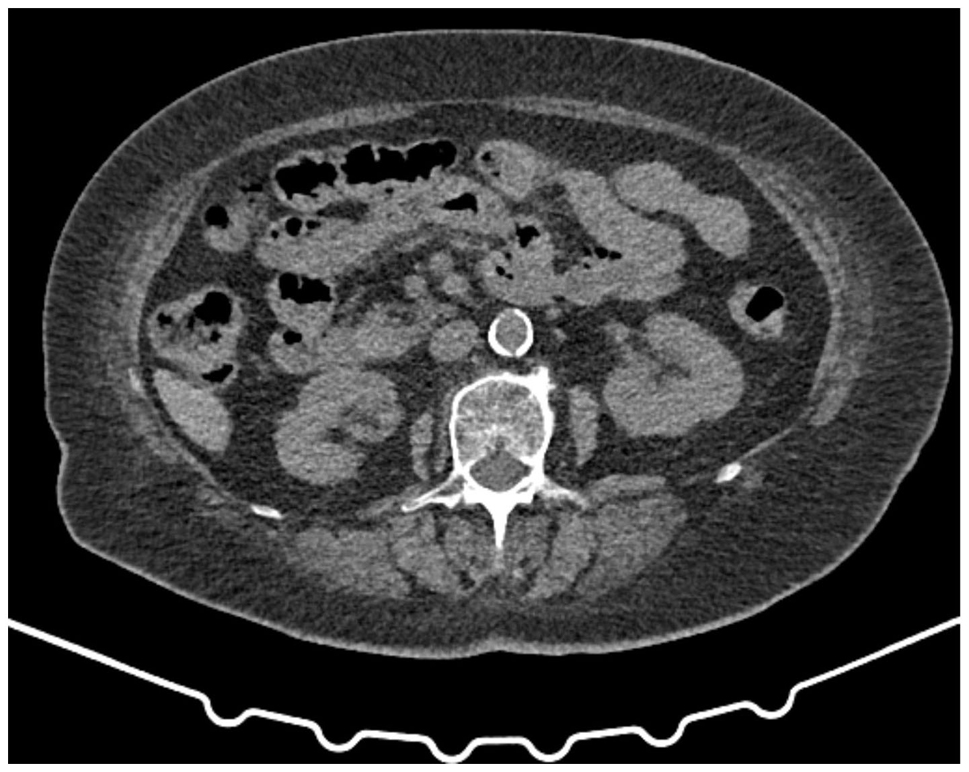
Lower sections of the thoracic computed tomography scan revealed no abnormalities in the renal parenchyma and collecting system.

The results of laboratory tests are presented in Table I. An abdominal ultrasonographic examination revealed grade 1 renal parenchymal disease without obstruction. Bilateral pleural fluid, cardiomegaly and metallic suture and clip secondary to CABG and SAVR were determined in the chest X-ray and the lower sections of the thoracic computed tomography scan revealed no abnormalities in the renal parenchyma and collecting system (Figs. 1 and 2). Echocardioraphic measurements are presented in Table II.

### Clinical management

Anti-hypertensive medications were discontinued. The systolic blood pressure was targeted to be 120-150 mmHg. Hyponatremia was asymptomatic and secondary to hypervolemia and dilutional. Intravenous (IV) furosemide at 300 mg/day and oral isosorbid-5-mononitrate at 20 mg/day were initiated. Warfarin was continued as 1x1.25 mg/day according to target level an international normalized ratio (INR) of 2-3. The insulin dose was adjusted to maintain blood sugar levels at 110-150 mg/dl. As regards blood gases, the pH and HCO_3_ were >7.3 and >19, respectively. The patient had nonoliguric AKI. Oral calcium acetate was administered for hyperphosphatemia and hypocalcemia, and IV calcium infusion was administered. On the 14th day of hospitalization, the serum levels of BUN (107 mg/dl) and creatinine (11 mg/dl) began to decrease. Dialysis was not performed and she was discharged on day 15. During an outpatient clinical follow-up, the patient was asymptomatic and her blood pressure was 120/70 mmHg. At the last visit (45 days after the onset of acute renal failure and 97 days after CABG/SAVR), the glomerular filtration rate was 47.3 ml/min. No proteinuria, pyuria, or cell casts were detected.

## Discussion

The present case report described the case of a 76-year-old diabetic woman with AKI and hypertension who underwent CABG and SAVR. In addition to complications related to surgery, malnutrition and poor physical performance also affect prognoses. Despite adverse factors, invasive approaches should not be avoided in the treatment of ischemic heart disease-myocardial infarction, and complete revascularization is recommended in the presence of multivessel disease (6).

### AKI and cardiovascular surgery in the elderly

A critical issue among elderly patients undergoing cardiovascular surgery is the development of AKI in the post-operative period. In the early period following SAVR, AKI lasting <48 h has been reported in 45.1% of elderly patients, while persistent AKI has been found in 18.6% of patients (7). Independent risk factors for persistent AKI are severe heart failure (New York Heart Association, Grade III-IV), moderate renal dysfunction, anemia and AKI (stages 2 and 3). Complications are more common in patients with AKI. Major adverse events in the hospital occur at a rate of 3.9% in those without AKI, 4.7% in patients with early-recovery AKI, and 20.6% in patients with persistent AKI (7). The patient described herein did not develop AKI in the early post-operative period; however, AKI stage 3 developed at 2 months following surgery, and resolved without the loss of the glomerular filtration rate (GFR). AKI is staged using criteria that assess changes in the serum creatinine as follows: Stage 1, an increase in the baseline serum creatinine levels (bSCr) by 1.5-1.9-fold; stage 2, an increase in bSCr by 2-2.9-fold; stage 3, an increase in bSCr by 3-fold, or an increase in serum creatinine levels to >4.0 mg/dl or the initiation of renal replacement therapy (8).

In a previous study comparing to 190 patients <50 years of age and 388 patients >80 years of age who underwent CABG, 20.9% of the older patients experienced at least one post-operative complication compared to 10% of the younger patients (9). Among the patients >80 years, hypertension and DM were more common. In that study, major adverse cardiac and cerebrovascular events (MACCE) were more common among elderly patients with advanced heart failure and cerebrovascular accidents. Mortality occurred in 79.4% of the elderly patients who developed MACCE. In patients with a high ejection fraction, MACCE was lower. As regards post-operative complications in patients <50 years of age, the risk factor was emergency admission (9). In the patient in the present study, MACCE did not develop following combined CABG and SAVR.

Pre-operative neurocognitive function is a key factor affecting complications and the length of hospitalization following transcatheter aortic valve replacement in elderly patients (10). The pre- and post-operative neurological condition of the patient described herein was good.

### Oral anticoagulants and AKI in patients undergoing valve replacement

Bleeding is a critical issue in valve replacement patients due to the anticoagulants. In the patient described herein, a hematoma and abscess developed at the incision site in the lower sternum in the early post-operative period and rapidly resolved with drainage and antibiotic therapy. The type of anticoagulants used in patients undergoing bioprosthetic valve replacement is critical. Compared to warfarin, new oral anti-coagulants (NOACs) were found to be safer in the REVERSE study (11). Rivaroxaban was used for early post-operative anticoagulation in patients undergoing bioprosthetic valve surgery for 6 months in the REVERSE trial. Rivaroxaban, an NOAC, was found to be safe and effective compared to warfarin. Warfarin was prescribed as an anticoagulant (11). Early post-operative period bleeding developed in the patient in the present study. The dose was adjusted to target an INR of 2-3 in the patient described herein, and bleeding was not observed at a later period. Although bleeding was considered as a potential cause of AKI, urine analysis and imaging (Fig. 2) did not support this etiology. Another etiology of AKI may have been acute interstitial nephritis (AIN) due to incision site abscess, infection and the drugs/antibiotic(s) used in the post-operative period. However, proteinuria, eosinophilia and pyuria, as well as kidney failure supporting a diagnosis of AIN, were not detected in the patient in the present study. The history of hypotension at home following discharge and continuing cilazapril (5 mg), hydrochlorothiazide (12.5 mg) (ACEI) therapy during this period suggested hemodynamic AKI/acute tubular necrosis (ATN) in the patient described herein. An older age, hypotension due to anti-hypertensive therapy and heart failure may be considered predisposing factors for ATN. During the AKI (ATN), a urine test revealed a density of 1.005 and a few erythrocytes (after urinary catheterization) with traces of protein. At the last out-patient clinic visit, the urine density of the patient was 1.020, and the absence of proteinuria and hematuria support the possibility that AKI/ATN had improved.

Changes in renal function are critical for making prognoses in elderly patients undergoing valve replacement surgery. In a previous study, in 4,531 patients >60 years of age, a post-operative GFR decrease of >50% compared to pre-operative GFR was an independent risk factor for hospital mortality and all-cause mortality at the 1-year follow-up (12).

Patients with chronic kidney disease (CKD) requiring cardiovascular surgery have a high comorbidity, similar to elderly patients. In a previous study, renal function remained stable or improved after 1 month in >80% of patients with CKD who underwent transcatheter valve replacement (13). In addition, the 2-year mortality was lower in patients with improved renal function. Mortality was found to be higher in patients with deteriorating renal function. Therefore, in patients requiring transcatheter aortic valve implantation (TAVI), cardiorenal syndrome may be a possible cause of CKD, and cardiac and renal function may improve after the procedure (13).

### SAVR or TAVI

SAVR in elderly patients is controversial. In the present study, the 76-year-old patient underwent a surgical bioprosthetic heart valve replacement. In a previous study, TAVI was more advantageous in the early post-operative period when comparing TAVI and redo-aortic valve replacement (redo-AVR) in terms of prognosis in patients with bioprosthetic valve insufficiency 14). Hospital mortality was 7.2% in the redo-AVR group and 0% in the TAVI group. The need for intra-aortic balloon pump support, early re-operation, respiratory and neurological complications, and multiorgan failure were higher in the surgical group. The TAVI group had a shorter period of intensive care and hospitalization, but moderate aortic leakage and postprocedural gradients at discharge. In patients discharged from the hospital after successful procedures, the prognosis at the 6-year follow-up was similar after both TAVI and SAVR (14).

In another study, in elderly patients with aortic stenosis (AS) who underwent SAVR with partial mini-sternotomy, the quality of life was lower compared to patients who underwent transfemoral TAVI (15). However, patients who underwent SAVR with partial upper mini-sternotomy had improved technical outcomes, with fewer pacemaker implantations and less paravalvular leakage. SAVR required more blood transfusions, longer ventilation and a longer period of hospitalization in the intensive care unit (15). The patient in the present study was hospitalized due to the development of a hematoma and abscess at the sternotomy site following SAVR. In patients with AS and end-stage renal disease, as in numerous elderly patients, TAVI has been shown to be associated with a lower hospital mortality, fewer complications, shorter periods of hospitalization, lower treatment costs, and a higher rate of discharge to home compared to SAVR despite the increased comorbidities and disease burden (16). In another study, in the SAVR group with chronic heart failure and symptomatic AS, the comorbid burden was higher compared to the TAVI group, although hospital mortality was similar (17). However, cardiovascular, respiratory and renal issues were less common in patients who underwent TAVI. Therefore, TAVI can be considered safer in these patients (17).

A significant association was previously found between increased serum creatinine levels and the prognosis of patients who underwent SAVR (18). In a long-term follow-up, all-cause mortality was similar in patients with no or minimal increases in early serum creatinine. The 30-day mortality was increased in patients with increased serum creatinine levels. The risk of developing heart failure and chronic renal failure was minimally increased (18). Quality of life was found to be improved in elderly patients who underwent SAVR and the risk of reoperation due to structural degeneration was lower. Mortality was increased in non-valvular noncoronary procedures combined with SAVR (19).

### Isolated aortic valve replacement or valve replacement combined with coronary artery surgery

In a previous study, in patients >75 years with isolated SAVR, the duration of hospitalization and mechanical ventilation, the need for dialysis, and the mortality were lower in the early period compared to combined CABG and SAVR (20). Long-term mortality did not differ between the groups (20).

### Predictive score for the outcomes of cardiovascular surgery

In a previous study, pre-operative EUROSCORE and Thoracic Surgeon risk scores were not found to be useful in assessing postoperative complications and prognosis in elderly patients undergoing SAVR (21).

### Mechanical or bioprosthetic valve replacement

An SAVR bioprosthetic valve was used in the patient in the present study. Compared to the outcome for bioprosthetic valves, mechanical valve implantation in the 50-65 age group has demonstrated improved results for long-term survival with fewer major adverse events and fewer re-operations required (22).

### Invasive or conservative treatment in the elderly with coronary ischemic heart disease

In the ISCHEMIA trial, 1,236 patients with three-vessel coronary artery disease were evaluated (23). The revascularization group [612 patients; invasive group (CABG/percutaneous intervention)] was compared with the conservative group (624 patients; medical treatment), and cardiovascular mortality and myocardial infarction was found to be less common, while quality of life was improved over a period of 4 years in the invasive group. The differences in all-cause mortality rates between revascularization and conservative were, however, small, with wide confidence intervals (23).

### Hypertension treatment

It should be remembered that hypotension can develop more frequently in the treatment of hypertensive elderly patients. Hypotension may impair cerebral and renal perfusion due to arterial stiffness, leading to AKI and other complications. OH has been reported at a rate of 7-10% in hypertensive adults, especially in older patients, and is also associated antihypertensive drugs (5). In the patient described herein, standard antihypertensive therapy caused hypotension and AKI, falls and ecchymosis. It is thus critical to measure blood pressure at home, and patients and their family need to be informed what the target blood pressure level is. Patients should be encouraged to contact their physician if they experience abnormal blood pressure values. Preventive measures such as good control of blood pressure can reduce complications. In a previous study, the most common adverse events among 6,580 patients, including 1,237 patients with heart failure with reduced ejection fraction treated with four drugs were hypotension, AKI (mean 28 days) and hyperkalemia (24).

Renin-angiotensin system inhibitors are recommended as the first-line treatment for hypertension in the elderly (25). In one cohort, severe side-effects were observed with anti-hypertensive therapy; however, the absolute risk was low, apart from elderly patients with moderate or severe frailty (26). The likelihood of benefit from treatment in this group of patients was similar to the risks of hypotension, falls, AKI, electrolyte disturbances and syncope. Therefore, physicians may avoid prescribing new medications and consider alternative approaches when treating these patients (26).

In general, stringent blood pressure control improves cardiovascular outcomes in hypertensive patients. In the majority of subpopulations, intensive blood pressure control is preferred over less intensive blood pressure control, although the data for patients with diabetes or cardiovascular disease are less clear (27). It is not recommended to overtreat hypertension in elderly patients (28). In the patient in the present study, AKI stage 3 developed secondary to hypotension. In critically ill elderly patients, the added stress factor of age-related structural deterioration and diminished renal reserve increases susceptibility to renal function deterioration. Age is an independent risk factor for the development of AKI. Therefore, physicians should consider changes in renal function in elderly patients and implement preventive measures (29).

### Arrhythmia during cardiovascular surgery

The patient in the present study developed atrial fibrillation (AF) in the post-operative period. AF is the most common arrhythmia that develops following cardiac surgery. Cardiopulmonary bypass disrupts systemic homeostasis by triggering oxidative stress and ischemia-reperfusion injury, an inflammatory cascade. Prolonged aortic cross-clamping >60-75 min further triggers myocardial ischemia and structural remodeling, increasing the risk of developing post-operative AF (30). Post-operative AF following isolated CABG surgery has been reported at a frequency of 30.94%. Risk factors for the development of AF include an advanced age, hypertension, smoking, coronary bypass time and a low EF (31). In the patient described herein, AF was controlled with metoprolol.

In conclusion, mortality and morbidity are increased in elderly patients following cardiovascular surgery, and periods of hospitalization are longer. Invasive approaches should not be avoided in the treatment of ischemic heart disease or myocardial infarction, and complete revascularization is recommended in the presence of multi-vessel disease. Although early mortality is high in patients undergoing CABG and SAVR, the long-term prognosis is similar to that of patients who undergo single SAVR and combined surgery. According to the REVERSE study (11), NOACs are recommended for patients requiring post-operative anticoagulants. Notably, the patient in the present study developed hypotension and acute renal failure related to standard anti-hypertensive drugs. Due to the increased risk of renal failure in elderly patients, it is important to avoid hypotension and implement preventive measures. The overtreatment of hypertension in the elderly should be avoided.

In general, there is a need for greater caution regarding hypotension and OH in the treatment of hypertension in elderly patients, particularly those with comorbid conditions. Clinicians should note that while high blood pressure is dangerous, excessively low blood pressure can also cause complications, such as falls, AKI and hospitalization. It is important that blood pressure management be individualized in accordance with guidelines. Patients should be encouraged to measure their blood pressure at home, and written blood pressure target values should be provided to both the patient and their caregivers.

## Figures and Tables

**Table I:** Results of hematological and renal function tests during the follow-up period.

Parameters	Pre-operative	A: Day 61; B: Day 5	A: Day 65; B: Day 8	A: Day 69; B: Day 14	A: Day 74; B: Day 19	A: Day 78; B: Day 23	A: Day 82; B: Day 29	A: Day 97; B: Day 45
Hb (g/dl)	10.9	10.3	8.6	8.6	8.5		9.5	10.3
WBC (cells/cumm)	9,180	9,330	7,380	6,730	6,890		6,490	5,990
BUN (6-20 mg/dl)	18	114	126	103	98	69	42	18
Creatinine (0.55-1.02 mg/dl)	0.92	10.30	10.00	10.00	9.14	5	2.39	1.13
Sodium (136-145 mEq/l)	143	126	124	136	138	143	141	143
Potassium (3.5-5.1 mEq/l)	4.4	5.28	5.67	4.78	3.93	4.62	3.56	4.13
Calcium (8.6-10 mg/dl)	9.84	5.94	5.83	7.29	7.63	7.61	8.34	
Phosphorus (2.5-4.9 mg/dl)		11	11.40	6.61	7.29	3.48	3.14	
PT INR (0,8-1.25)	1.09	1.63	2.40	2.40	2.88	2.88	1.83	
Blood pressure mmHg	130/70	117/67	106/68	115/58	114/70	125/72		
Pulse per minute		60	70	102	60	70		
Urine volume day (ml)		1,200	1,250	2,750	1,250	1,600		
Urine density	1.030	1.005	1.005	1.015				1.020

A, post-operative day; B, the day following hypotension; Hb, hemoglobin; WBC, white blood cells; BUN, blood urea nitrogen; PT, prothrombin time; INR, international normalized ratio.

**Table II:** Echocardiographic measurements of the patient.

Parameters	March 14, 2025; pre-operative day 5	May 12, 2025; post-operative day 54	June 25, 2025; post-operative day 98
Left atrium (mm)	43	46	16
Interventricular septum (mm)	14	15	13
Left ventricular posterior wall thickness (mm)	13	14	11
Left ventricular ejection fraction (%)	68	70	71
Aortic mean gradient (mHg)	4,6	7	5
Aortic peak gradient (mmHg)	4-6	7	5
Pulmonary arterial pressure		38	

## Data Availability

The data generated in the present study may be requested from the corresponding author.
